# Supplementary material for: Distribution of Phototrophic Purple Nonsulfur Bacteria in Massive Blooms in Coastal and Wastewater Ditch Environments
Source: Microorganisms. 2020 Jan 22;8(2):150. doi: 10.3390/microorganisms8020150 (PMC7074854; doi:10.3390/microorganisms8020150)
Supplement: Supplementary file 1 [file microorganisms-08-00150-s001.pdf]

Supplementary materials

Article

Distribution of Phototrophic Purple Nonsulfur Bacteria in Massive Blooms in Coastal and Wastewater Ditch Environments

Akira Hiraishi [1,*], Nobuyoshi Nagao [1], Chinatsu Yonekawa [1], So Umekage [1], Yo Kikuchi [1], Toshihiko Eki [1,2], and Yuu Hirose [1,2,*]

[1] Department of Environmental and Life Sciences, Toyohashi University of Technology, Toyohashi 441-8580, Japan; velvetschild@gmail.com (N.N.); hgm.cnt@gmail.com (C.Y.); soumekage@gmail.com (S.U.); kikuchi@tut.jp (Y.K.)

[2] Department of Applied Chemistry and Life Science, Toyohashi University of Technology, Toyohashi 441-8580, Japan; eki@chem.tut.ac.jp (T.E.)

*Correspondence: hiraishi@ens.tut.ac.jp (A.H.); hirose@chem.tut.ac.jp (Y.H.)

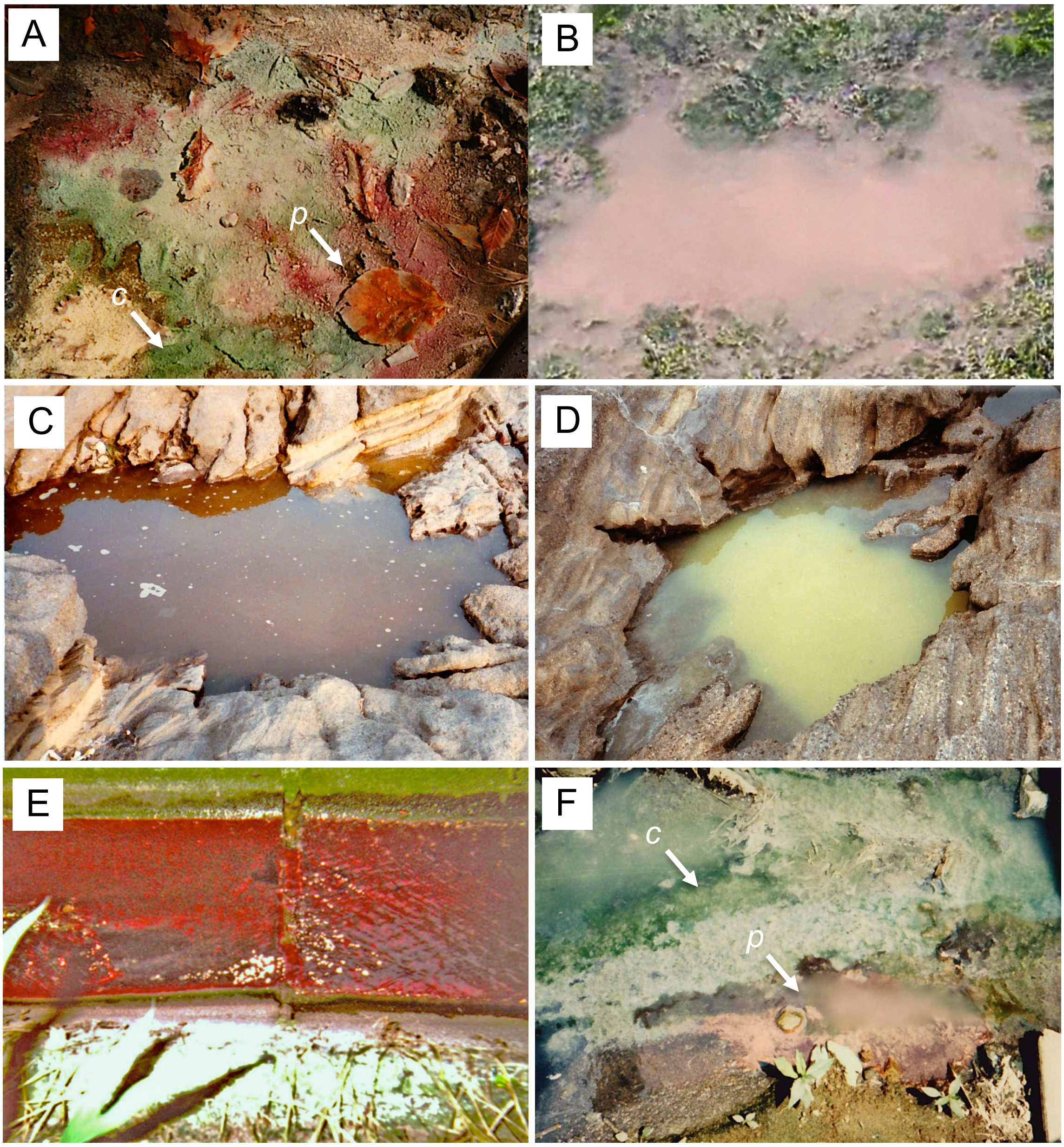

Figure S1. Appearance of colored blooms and microbial mats studied. A, hot spring H1 (red); B, mud flat Y1 (pink); C, tide pool J2 (red-brown), D, tide pool J3 (yellow-green); E, wastewater ditch D1 (red); F, sewage ditch D3 (pink). For H1 and D3, the dual blooms/mats of cyanobacteria and purple bacteria are shown by arrows *c* and *p*, respectively.

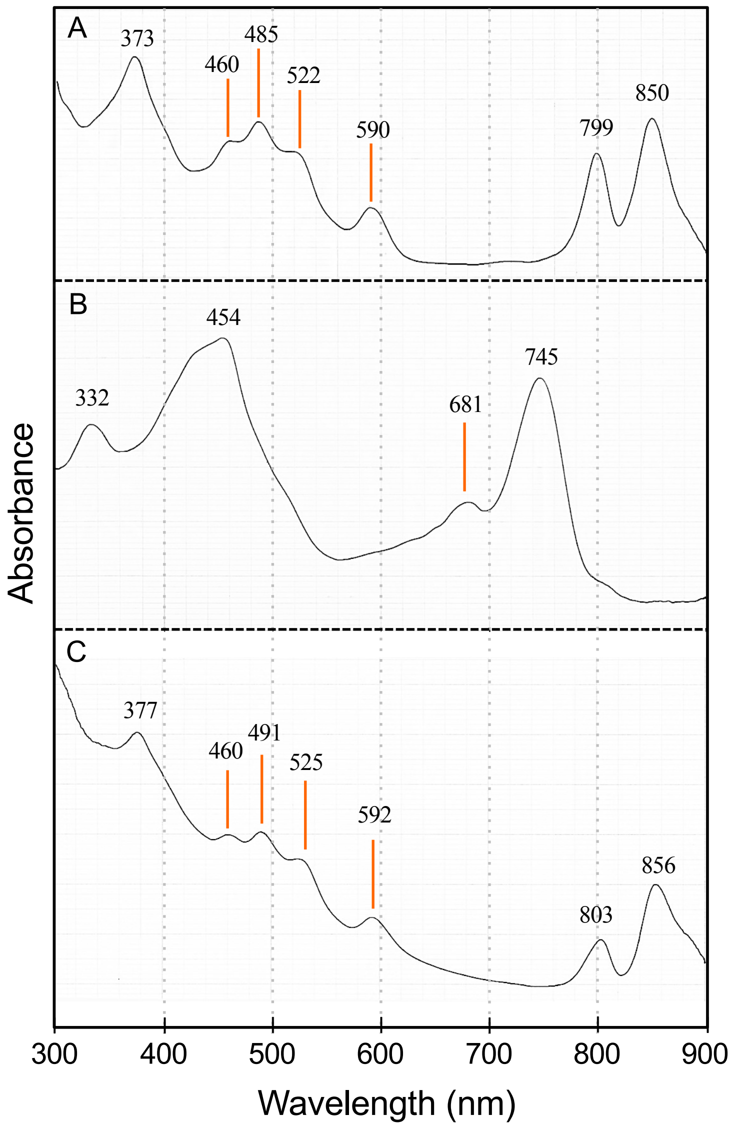

Figure S2. *In vivo* absorption spectra of the biomass collected from pink mud flat Y1 (A), yellow-green tide pool J3 (B), and red ditch mat D3 (C).

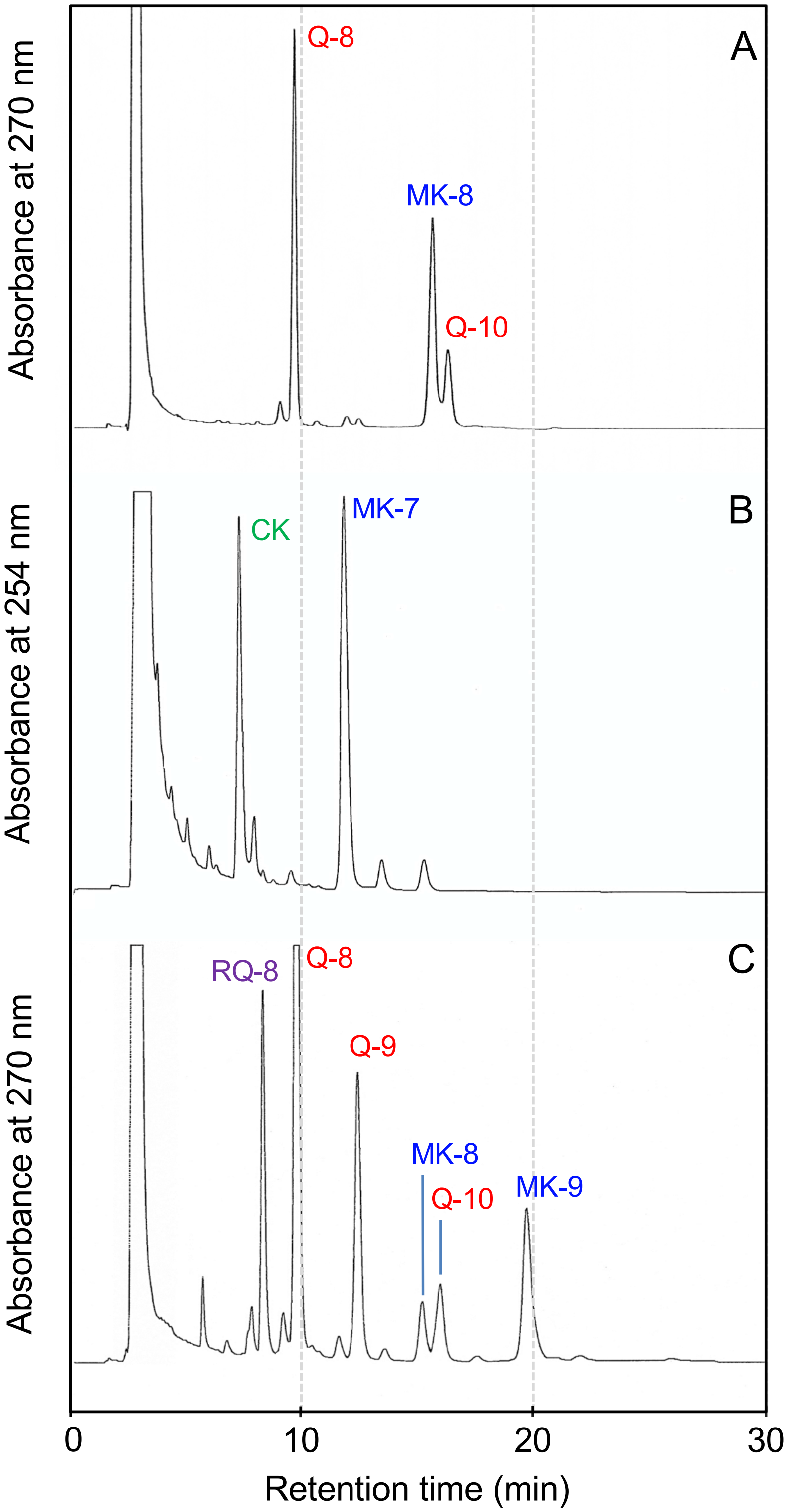

Figure S3. HPLC elution profiles of isoprenoind quinones extracted from pink mud flat Y1 (A), yellow-green tide pool J3 (B), and red ditch mat D3 (C). Main quinone homologs identified are shown at the top of respective peaks by different colors of letters: red, ubiquinones; purple, rhodoquinone-8; blue, menaquinones; green, chlorobiumquinone.

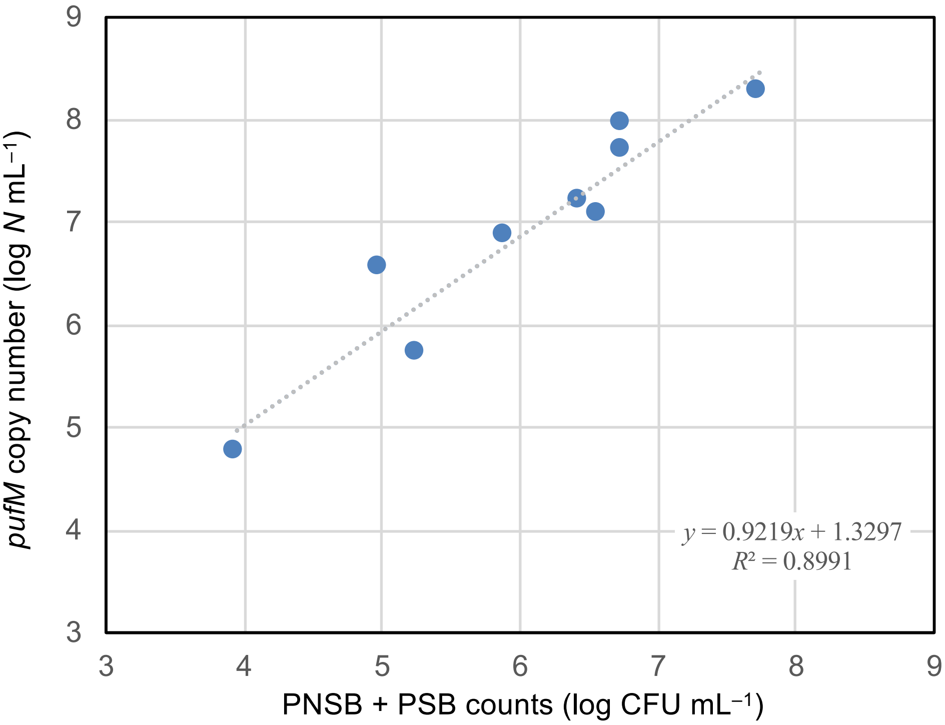

Figure S4. Relationship between *pufM* gene copy numbers and viable counts of PNSB + PSB in colored blooms and mats investigated. A deduced regression equation and the correlation coefficient are given in the plot. The correlation is significant at $p < 0.001$.

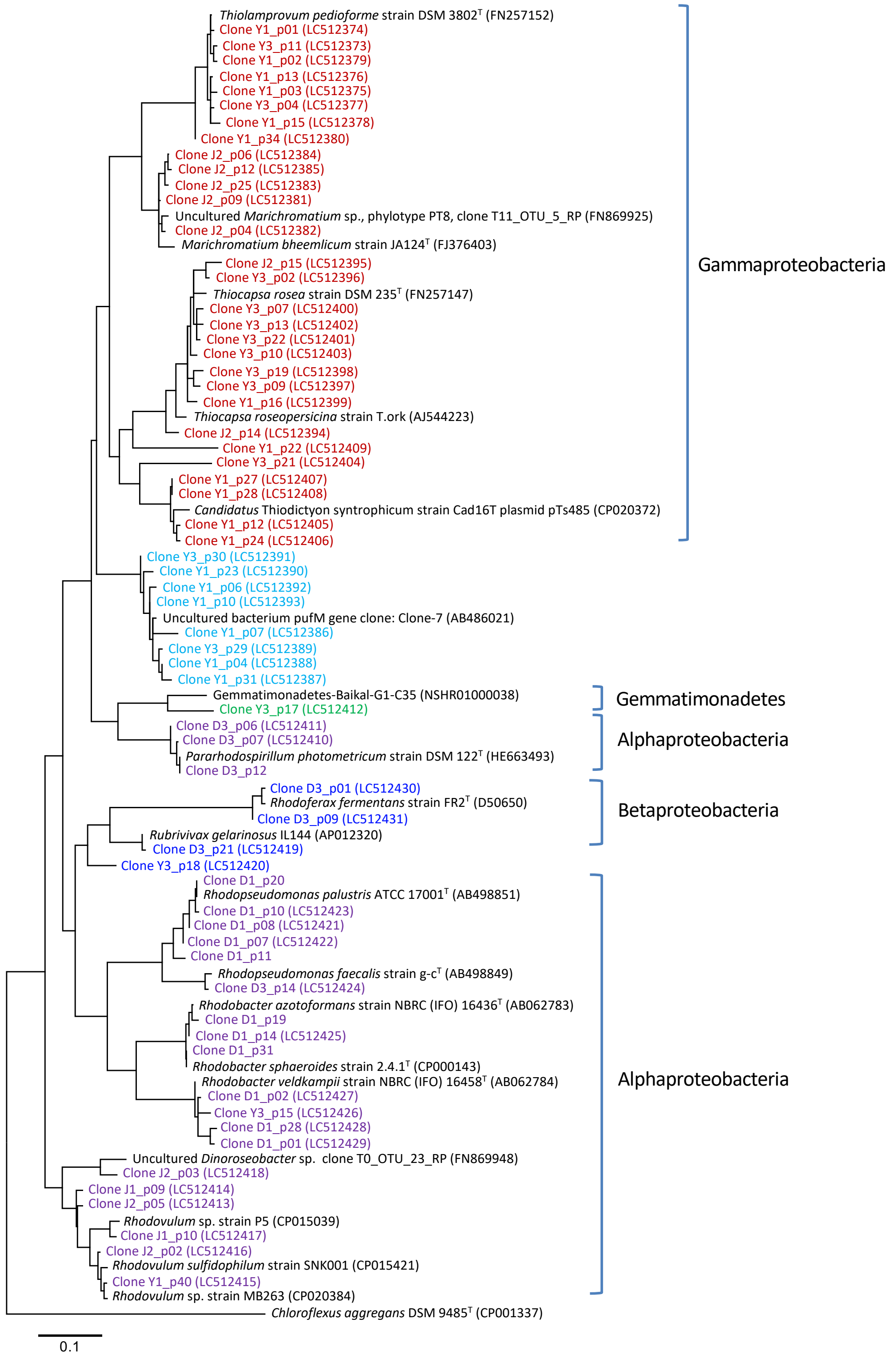

Figure S5. Maximum-likelihood phylogenetic tree of the *pufM* gene clones detected and their closest relatives. The clones of *Gammaproteobacteria*, *Gemmatimonadetes*, *Alphaproteobacteria*, *Betaproteobacteria*, and an unassigned phylogenetic group are shown by brown, green, blue, purple, and light blue letters, respectively. The database accession numbers for the sequences incorporated are shown in parentheses behind clone and organism names. *Chloroflexus aggregans* DSM 9485^T was used as an outgroup to root the tree. Scale bar = 0.1 substitution per position..

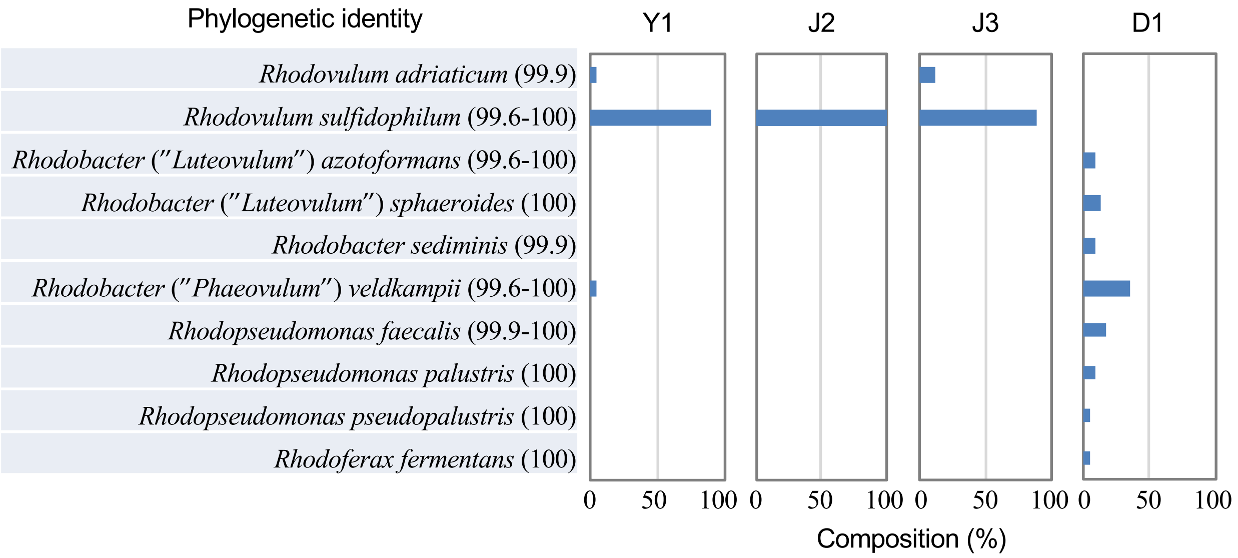

Figure S6. 16S rRNA gene sequence-based phylogenetic composition of the PSNB isolates from red mud flat J1, red tide pool J2, yellow-green tide pool J3, and red ditch mat D1. The numbers of isolates investigated were 18 for Y1, 19 for J1, 18 for J3, and 23 for D1. The phylogenetic identity was determined by EZbioCloud homology search. Figures in parentheses show sequence similarity levels (%) to the type strains of respective species.

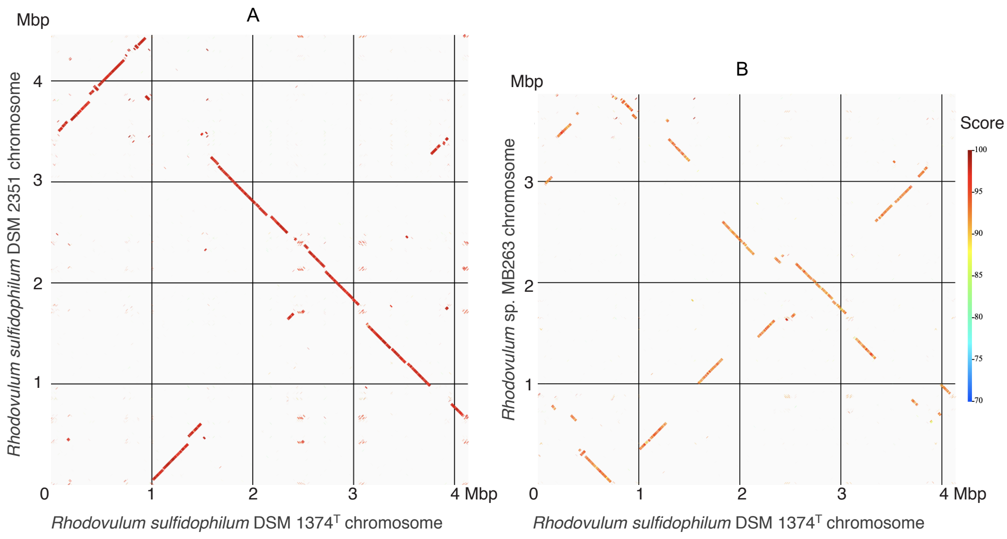

Figure S7. Harr plots showing genomic similarities between *Rhodovulum sulfidophilum* strains DSM 1374^T and DSM 2351 (A) and between *Rhodovulum sulfidophilum* strain DSM 1374^T and *Rhodovulum* sp. strain MB263 (B).

Table S1. PCR primers used for specific gene amplification in this study.

Primer	Target gene	Direction	Sequence (5'→3')	Reference*
27f	16S rRNA	Forward	AGAGTTTGATCMTGGCTCAG	Lane [37]
1492r	16S rRNA	Reverse	TACGGYTACCTTGTTACGACTT	Lane [37]
357f	16S rRNA	Forward	CCTACGGGAGGCAGCAG	Lane [37]
519r	16S rRNA	Reverse	GWATTACCGCGGCKGCTG	Lane [37]
pufM.557mF	pufM	Forward	TTCCCSCACCTSGAYTGGAC	This study
pufM.750mR	pufM	Reverse	CCCATSGTCCARCGCCAGAA	This study
M151f	pufM	Forward	CGCACCTGGACTGGAC	Okubo et al. [19]

* The reference numbers correspond to those appearing in the Reference list in the article.

Table S2. Similarity levels of 16S rRNA gene and whole genome sequences between *Rhodovulum* sp. strain MB263 (accession CP020384.1) and authentic strains of established *Rhodovulum* species.

Species and strain	16S rRNA gene sequence		Genome	
	Accession	Identity (%)	Accession	ANI score (%)
Rhv. sulfidophilum DSM 1374^T	DF260912	99.6	NZ_CP015418	91.21
Rhv. sulfidophilum DSM 2351	AP014800	99.6	NZ_AP014800	91.11
Rhv. algae JA877^T	LN908891	99.6	NA*	NT†
Rhv. visakhapatnamense JA181^T	AM180707	98.9	NZ_SOEB01000001	87.84
Rhv. viride JA756^T	HE983843	98.7	NZ_MUAV01000001	86.94
Rhv. kholense DSM 19783^T	AM748927	97.4	NZ_QAYC01000001	87.43

* No information available.
† Not tested.

Table S3. List of *Rhodovulum* species and strains and the accession numbers for genome sequences used for reconstruction of the phylogenomic tree based on 92 core protein-coding gene sequences. The strains whose genome sequences were determined in this study are shown by red letters.

Species	Strain	Accession and version	Reference*
Rhodovulum adriaticum	DSM 2781^T	NZ_SLXL00000000.1	Unpublished
Rhodovulum bhavnagarense	DSM 24766^T	NZ_SLXU01000001.1	Unpublished
Rhodovulum euryhalinum	DSM 4686^T	NZ_SLWW01000001.1	Unpublished
Rhodovulum imhoffii	DSM 18064^T	NZ_QAAA01000001.1	Unpublished
Rhodovulum kholense	DSM 19783^T	NZ_QAYC01000001.1	Unpublished
Rhodovulum marinum	DSM 18063^T	NZ_SLXP01000001.1	Unpublished
Rhodovulum robiginosum	DSM 12329^T	NZ_RWGU01000051.1	Unpublished
Rhodovulum steppense	DSM 21153^T	NZ_SLVM01000001.1	Unpublished
Rhodovulum strictum	DSM 11289^T	NZ_WJPO00000000.1	Unpublished
Rhodovulum sulfidophilum	DSM 1374^T	NZ_CP015418.1	This study
Rhodovulum sulfidophilum	DSM 2351	NZ_AP014800.1	Nagao et al. [63]
Rhodovulum sulfidophilum	AB14	NZ_MSYP00000000.1	Unpublished
Rhodovulum sulfidophilum	AB26	NZ_MSYQ00000000.1	Unpublished
Rhodovulum sulfidophilum	AB30	NZ_MSYR00000000.1	Unpublished
Rhodovulum sulfidophilum	S2_005_002_R2_34	QFPW00000000.1	Brooks et al. [69]
Rhodovulum sulfidophilum	SNK001	NZ_CP015421.1	Unpublished
Rhodovulum viride	JA756^T	NZ_MUAV01000001.1	Unpublished
Rhodovulum visakhapatnamense	JA181^T	NZ_SOEB00000000.1	Unpublished
Rhodovulum sp.	12E13	NZ_QPLK00000000.1	Unpublished
Rhodovulum sp.	BSW8	NZ_QNVX00000000.1	Unpublished
Rhodovulum sp.	MB263	NZ_CP020384.1	This study
Rhodovulum sp.	NI22	NZ_JQFU00000000.1	Brown et al. [70]
Rhodovulum sp.	P5	NZ_CP015039.1	Unpublished
Rhodovulum sp.	PH10	NZ_AKZI00000000.1	Khatri et al. [71]

* The reference numbers correspond to those appearing in the Reference list in the article.
